# Supplementary material for: The impact of long-term care needs on the socio-economic deprivation of older people and their families: A scoping review protocol
Source: PLoS One. 2022 Aug 31;17(8):e0273814. doi: 10.1371/journal.pone.0273814 (PMC9432749; doi:10.1371/journal.pone.0273814)
Supplement: S1 Checklist — (DOCX) [file pone.0273814.s001.docx]

**PRISMA-P (Preferred reporting items for systematic review and meta-analysis protocols**) **2015 checklist: recommended items to address in a systematic review protocol**

| **Section/topic** | **#** | **Checklist item** | **Information reported** | | | **Line number(s)** |
| --- | --- | --- | --- | --- | --- | --- |
|  |  |  | **Yes** | | **No** |  |
| **ADMINISTRATIVE INFORMATION** | | | | | | |
| **Title** | | | | | | |
| Identification | 1a | Identify the report as a protocol of a scoping review | • |  | | 4; 12 (and Title) |
| Update | 1b | If the protocol is for an update of a previous systematic review, identify as such |  | • | | N/A |
| **Registration** | 2 | If registered, provide the name of the registry (e.g., PROSPERO) and registration number | • |  | | 151-154 |
| **Authors** | | | | | | |
| Contact | 3a | Provide name and institutional affiliation of all protocol authors; provide the e-mail address of the corresponding author | • |  | | Title page |
| Contributions | 3b | Describe contributions of protocol authors and identify the guarantor of the review | • |  | | 359-365 |
| **Amendments** | 4 | If the protocol represents an amendment of a previously completed or published protocol, identify as such and list changes; otherwise, state plan for documenting important protocol amendments |  | • | | N/A |
| **Support** | | | | | | |
| Sources | 5a | Indicate sources of financial or other support for the review | • |  | | See note 1 |
| Sponsor | 5b | Provide name for the review funder and/or sponsor | • |  | | See note 1 |
| Role of sponsor/funder | 5c | Describe roles of funder(s), sponsor(s), and/or institution(s), if any, in developing the protocol | • |  | | See note 2 |
| **INTRODUCTION** | | | | | | |
| **Rationale** | 6 | Describe the rationale for the review in the context of what is already known | • |  | | 1-12; 37-50 |
| **Objectives** | 7 | Provide an explicit statement of the question(s) the review will address with reference to participants, concepts, context (PCC) | • |  | | 168-200 |
| **METHODS** | | | | | | |
| **Eligibility criteria** | 8 | Specify the study characteristics (e.g., PICO, study design, setting, time frame) and report characteristics (e.g., years considered, language, publication status) to be used as criteria for eligibility for the review | • |  | | 201-214 |
| **Information sources** | 9 | Describe all intended information sources (e.g., electronic databases, contact with study authors, trial registers, or other grey literature sources) with planned dates of coverage | • |  | | 219-232 |
| **Search strategy** | 10 | Present draft of search strategy to be used for at least one electronic database, including planned limits, such that it could be repeated | • |  | | 139-144; 162-164; 260-265^3^ |
| ***STUDY RECORDS*** | | | | | | |
| Data management | 11a | Describe the mechanism(s) that will be used to manage records and data throughout the review | • |  | | 305-316 |
| Selection process | 11b | State the process that will be used for selecting studies (e.g., two independent reviewers) through each phase of the review (i.e., screening, eligibility, and inclusion in meta-analysis) | • |  | | 115-120; 123-130; 254-256 |
| Data collection process | 11c | Describe planned method of extracting data from reports (e.g., piloting forms, done independently, in duplicate), any processes for obtaining and confirming data from investigators | • |  | | 293-305 |
| **Data items** | 12 | List and define all variables for which data will be sought (e.g., PICO items, funding sources), any pre-planned data assumptions and simplifications | • |  | | See note 4 |
| **Outcomes and prioritization** | 13 | List and define all outcomes for which data will be sought, including prioritization of main and additional outcomes, with rationale | • |  | | 317-325 |
| **Risk of bias in individual studies** | 14 | Describe anticipated methods for assessing risk of bias of individual studies, including whether this will be done at the outcome or study level, or both; state how this information will be used in data synthesis | • |  | | 343-349 |
| ***DATA*** | | | | | | |
| **Synthesis** | 15a | Describe criteria under which study data will be quantitatively synthesized | • |  | | 305-316 |
|  | 15b | If data are appropriate for quantitative synthesis, describe planned summary measures, methods of handling data, and methods of combining data from studies, including any planned exploration of consistency (e.g., *I* ^2^, Kendall’s tau) | • |  | | 319-322 |
|  | 15c | Describe any proposed additional analyses (e.g., sensitivity or subgroup analyses, meta-regression) | • |  | | 322-325 |
|  | 15d | If quantitative synthesis is not appropriate, describe the type of summary planned |  | • | | N/A (326-329)^5^ |
| **Meta-bias(es)** | 16 | Specify any planned assessment of meta-bias(es) (e.g., publication bias across studies, selective reporting within studies) | • |  | | 334-339 |
| **Confidence in cumulative evidence** | 17 | Describe how the strength of the body of evidence will be assessed | • |  | | 243-249 |

^1^Information provided in the Financial Information section of the submission form, as indicated by the reference instructions for PLOS ONE submissions. ^2^Information provided in the Financial Information section of the submission form, as indicated by the reference instructions for PLOS ONE submissions. The funders had no role in study design, data collection and analysis, decision to publish, or preparation of the manuscript.

^3^Two dedicated tables were included in the main article (Tables 1-2).

^4^A specific data extraction tool was developed, i.e. a modified JBI data extraction instrument including all the variables involved (S1 Table).

^5^Both types of analyses were planned.
