# Supplementary material for: The impact of long-term care needs on the socio-economic deprivation of older people and their families: A scoping review protocol
Source: PLoS One. 2022 Aug 31;17(8):e0273814. doi: 10.1371/journal.pone.0273814 (PMC9432749; doi:10.1371/journal.pone.0273814)
Supplement: S1 Table — A, Study details and characteristics; B, Details or results to be extracted. (DOCX) [file pone.0273814.s002.docx]

| **A**) **Study Details and Characteristics** | | | | | | | | | | | | | | | | | | | | | | | | | | | | |
| --- | --- | --- | --- | --- | --- | --- | --- | --- | --- | --- | --- | --- | --- | --- | --- | --- | --- | --- | --- | --- | --- | --- | --- | --- | --- | --- | --- | --- |
| Study citation details  (e.g. authors; title; publication date) |  | | | | | | | | | | | | | | | | | | | YEAR | | | | | | | | |
|  |  |  |  |  |  |  |  |  |  |  |  |  |  |  |  |  |  |  |  |  | | | | | | | | |
| Study design  (recommendations and reporting guidelines that the authors refer to) |  | | | | | | | | | | | | | | | | | | | | | | | | | | | |
| Country  (where the study was conducted)  HIC = High Income Country; MIC = Middle Income Country; LIC = Low Income Country | NAME | | | | | | | HIC | | | | | | | MIC | | | | | LIC | | | | | | | | |
|  |  | | | | | | |  | | | | | | |  | | | | |  | | | | | | | | |
|  |  |  |  |  |  |  |  |  | | |  |  | | |  |  | |  | |  | | | | |  | | |  |
|  |  |  |  |  |  |  |  |  | | | | | | |  | | | | |  | | | | | | | | |
| Aims and keywords |  | | | | | | | | | | | | | | | | | | | | | | | | | | | |
| Type of study  (e.g. survey; secondary analysis of data from existing surveys) |  | | | | | | | | | | | | | | | | | | | | | | | | | | | |
| Participants  (e.g. family caregivers; nursing assistants) |  | | | | | | | | | | | | | | | | | | | | | | | | | | | |
| Sample type  (sampling methods; sample size) | not applicable | | | | | | | | not reported | | | | | | | | reported | | | | | | | | | | | |
|  |  | | | | | | | |  | | | | | | | |  | | | | | | | | | | | |
|  |  |  | |  | | | | |  | | | |  | |  | |  | | | | | | |  | | |  | |
|  |  | | | | | | | |  | | | | | | | |  | | | | | | | | | | | |
| Context  (e.g. long-term care; informal care) |  | | | | | | | | | | | | | | | | | | | | | | | | | | | |
| Health problems  (the reason for long-term care) |  | | | | | | | | | | | | | | | | | | | | | | | | | | | |
|  |  | | | | | | | | | | | | |  | | | | | | | | | | | | | | |
| *Multidimensional* concept of “deprivation” |  | | | | Y | |  | | | | | | |  | | | | | N | |  | | | | | | | |
|  |  | | | | | | | | | | | | |  | | | | | | | | | | | | | | |
| Methodology  (e.g. quantitative or qualitative approach) |  | | | | | | | | | | | | | | | | | | | | | | | | | | | |
| Techniques  (e.g. type of statistical analysis or interviews) |  | | | | | | | | | | | | | | | | | | | | | | | | | | | |
| Strengths and limitations  (as specified in the text) |  | | | | | | | | | | | | | | | | | | | | | | | | | | | |
| **B**) **Details or results extracted from the study** | | | | | | | | | | | | | | | | | | | | | | | | | | | | |
| Self reported economic difficulties  (what patients or family caregivers claim) |  | | | | | | | | | | | | | | | | | | | | | | | | | | | |
| Measurements  (e.g. the odds of having catastrophic health expenditure) |  | | | | | | | | | | | | | | | | | | | | | | | | | | | |
| Perspective on the relationship between socio-economic deprivation  and health: the role of *health conditions* and related variables | not applicable | | | | | | | | | dependent | | | | | | | independent | | | | | | | | | | | |
|  |  | | | | | | | | |  | | | | | | |  | | | | | | | | | | | |
|  |  | |  | | |  | | | |  | | |  | |  | |  | | | | | |  | | | |  | |
|  |  | | | | | | | | |  | | | | | | |  | | | | | | | | | | | |
| Perspective on the relationship between socio-economic deprivation  and health: the role of *economic conditions* and related variables | not applicable | | | | | | | | | dependent | | | | | | | independent | | | | | | | | | | | |
|  |  | | | | | | | | |  | | | | | | |  | | | | | | | | | | | |
|  |  | |  | | |  | | | |  | | |  | |  | |  | | | | | |  | | | |  | |
|  |  | | | | | | | | |  | | | | | | |  | | | | | | | | | | | |
| Perspective on the relationship between socio-economic deprivation  and health: the role of the *accessibility to formal care services* for the old | not applicable | | | | | | | | | dependent | | | | | | | independent | | | | | | | | | | | |
|  |  | |  | | |  | | | |  | | |  | |  | |  | | | | | |  | | | |  | |
|  |  | | | | | | | | |  | | | | | | |  | | | | | | | | | | | |
| Perspective on the relationship between socio-economic deprivation  and health: the role of the *type of assistance* and related variables | not applicable | | | | | | | | | dependent | | | | | | | independent | | | | | | | | | | | |
|  |  | |  | | |  | | | |  | | |  | |  | |  | | | | | |  | | | |  | |
|  |  | | | | | | | | |  | | | | | | |  | | | | | | | | | | | |
| Perspective on the relationship between welfare systems  and care-seeking behaviours: the role of the *type of welfare system* | not applicable | | | | | | | | | dependent | | | | | | | independent | | | | | | | | | | | |
|  |  | |  | | |  | | | |  | | |  | |  | |  | | | | |  | | | |  | | |
|  |  | | | | | | | | |  | | | | | | |  | | | | | | | | | | | |
| Reporting of limitations in activities of daily living  (the extent to which the patients to refer to are afflicted with limitations) | not applicable | | | | | | | | | not reported | | | | | | | reported | | | | | | | | | | | |
|  |  | |  | | |  | | | |  | | |  | |  | |  | | | | |  | | | |  | | |
|  |  | | | | | | | | |  | | | | | | |  | | | | | | | | | | | |
