# Supplementary figures and images for: The impact of long-term care needs on the socio-economic deprivation of older people and their families: A scoping review protocol
Source: PLoS One. 2022 Aug 31;17(8):e0273814. doi: 10.1371/journal.pone.0273814 (PMC9432749; doi:10.1371/journal.pone.0273814)

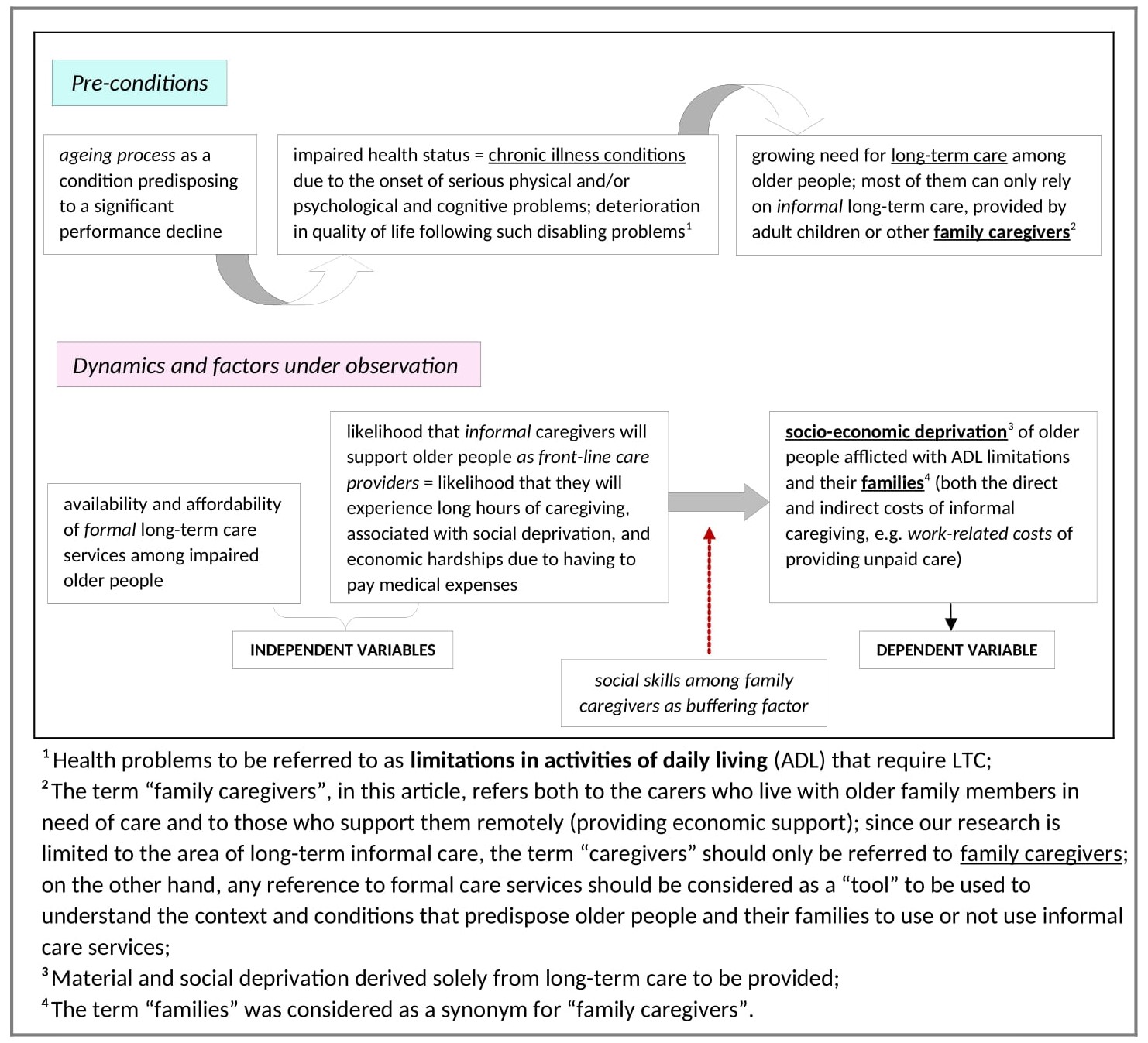

Supplement: S1 Fig — (JPG) [file pone.0273814.s003.jpg]
