# Supplementary material for: The impact of long-term care needs on the socio-economic deprivation of older people and their families: A scoping review protocol
Source: PLoS One. 2022 Aug 31;17(8):e0273814. doi: 10.1371/journal.pone.0273814 (PMC9432749; doi:10.1371/journal.pone.0273814)
Supplement: S2 Fig — (DOC) [file pone.0273814.s004.doc]

| **START**    Sketching out a list of initial research questions, e.g. what are the socio-economic conditions of families with older members to be provided with LTC? Do they experience socio-economic deprivation?    Identification of coherent general aims              NO  *Do these*  Are they too strictly defined?  If so, a systematic review (SR) should be done.  *aims meet the*  *ones specified by*  *Lockwood et al.*  *2019?*  **1**) identification of indicators of topics for subsequent systematic reviews;  **2**) identification of the ways in which studies address the issue/s; what concepts do they refer to?  **3**) identification of conceptual gaps  **(it should not be the sole purpose)**;  **4**) identification of the conceptual boundaries of the topic of interest;  **5**) identification/description of the types of the existing evidence;  **6**) identification of emerging findings;  **7**) orientation towards future studies  YES  If so [= they meet (most of) the seven aims listed by the authors], it is worth doing a scoping review (ScR)    Definition of the final research questions and related aims;  Definition of the first key-words (to start the search process)    Drafting of the protocol of the study  (description of all the stages of the ScR)    Description of the three main criteria for ScRs indicated by Lockwood et al. (2019): participants; concepts; context (PCC); description of the criteria to which the entire selection process refers (what studies should be sought?)  Listing of information concerning the ongoing study and all those selected (e.g. sources; evidence details; participants/concepts/context related to the selected studies); **explanation of the ways in which the existing relevant studies were conducted (relationship between the theories and the methods used)**      First selection  (35 selected articles)  Identification of the most  useful key-words    Second selection (a):  *Selecting selected articles*  Second selection (b):  *new selected articles*  Quality assessment and data extraction process (what type of analysis can be conducted?). **Structuring of the data extraction process**      Data analysis (qualitative analysis)  Data synthesis  **END**  Conclusions  YES  *Is it possible*  *to orient subsequent*  *systematic*  *reviews?*  NO |
| --- |
