# Supplementary material for: The impact of long-term care needs on the socio-economic deprivation of older people and their families: A scoping review protocol
Source: PLoS One. 2022 Aug 31;17(8):e0273814. doi: 10.1371/journal.pone.0273814 (PMC9432749; doi:10.1371/journal.pone.0273814)
Supplement: Attachment — Submitted filename: Response to Reviewers.docx [file pone.0273814.s006.docx]

**Plos One D-22-09758:** **The impact of long-term care needs on the socio-economic deprivation of older people and their families: A scoping review protocol**

**Reviewer 1**

| **n.** | **Reviewer comments** | **Authors’ answer** |
| --- | --- | --- |
|  | Thank you for asking me to review this protocol for a scoping review. The review aims to answer important questions and an appropriate method is described. | The authors are grateful for your interest and study. |
| **1** | Was the search strategy developed in conjunction with a research librarian? It would be useful to know this or why they were not involved. | Many thanks for your question. The study was conducted by an Italian team in which no research librarians were involved, as within the Italian context this position is not very common. However, in the section on keywords, we have specified that the keywords used have been carefully identified by a research team. This lack of research librarian support has been declared in the text in the *Conducting and reporting* section. |

**Reviewer 2**

| **n.** | **Reviewer comments** | **Line n. (based on the first version)** | **Authors’ answers** |
| --- | --- | --- | --- |
|  | **General comments** |  |  |
| 1 | Thank you for the opportunity to review this paper. I want to commend the authors on the focus of their study. This is a very interesting area for a review and could add new insights into the barriers to sufficient care of older adults. In general, a stronger scientific approach and more focused content is needed. From the outset it is unclear why a scoping review was chosen over a systematic review. |  | The authors are grateful to the reviewer for his/her detailed revision. |
| 2 | Language issues were highlighted in-text and I added suggestions could support you in clarifying the contents of the paper and strengthening its academic value. |  | All suggestions have been checked, evaluated and included in the text. |
|  | **Abstract** |  |  |
| 3 | Text changes proposed |  | Checked and accepted. |
|  | **Introduction** |  |  |
| 4 | What do these policies relate to? | 28 | The sentence has been changed to underline the relevance of LTC for welfare and health policy strategies. |
| 5 | Reconsider term used – not clear who this refers to | 30 | The term “actors” has been changed into “stakeholders”. |
| 6 | Reconsider word choice | 33 | The sentence has been revised to underline the impact of out-of-pocket expenditure on the financial availability of these families. |
| 7 | Purchase/require? | 34 | Changed word with “purchase”. |
| 8 | This is so true! A very tricky situation to navigate | 34 | Thanks, the authors appreciate your agreement. |
| 9 | Do you mean in both formal and informal care settings? | 49 | The sentence has been rewritten to improve the clarity of the concepts included and the readability of the text. |
| 10 | Do you mean the published evidence available? | 51 | The sentence has been rewritten to improve the clarity of the concepts included and the readability of the text. |
| 11 | related to(?) | 54 | Suggestion accepted by the authors: the sentence has been changed. |
| 12 | identify(?) | 55 | Suggestion accepted by the authors: the sentence has been changed. |
| 13 | what do you mean with this term? | 57 | A larger definition of multidimentional deprivation is included in the *Methods* section. However, the sentence has been revised and the main reference for the concept has been added. |
| 14 | Phrases and choice of words that need consideration will be highlighted in green | 60-64 | The sentences have been rewritten to better underline that the scoping review study focused on the multidimensional concept of socio-economic (SE) deprivation. |
| 15 | Introduce these concepts earlier on to guide the read | 73 | The reference to the multidimensional concepts of “family” and “poverty” has been introduced in the aims of the study (line 2), as previously suggested by you (suggestion 13). |
|  | **Methods** |  |  |
| 16 | Unclear whether you are introducing main guiding materials and when/where you will be discussing them? | 76 | We would like to introduce the main references of our methodological framework. The sentence has been changed to better clarify this aspect. |
| 17 | You have introduced the acronym before? | 77 | Yes, the sentence has been changed and only the acronym has been included in it. |
| 18 | So, the independent and depend on variable refer to chronic conditions? Consider the flow of your paper.  Consider to rewrite | 81 | The sentence has been changed to valorize that Fig. 1 shows the conceptual framework of the scoping review, useful to identify the involved variables. |
| 19 | Reconsider lay-out to let this paragraph follow on the first one in this section, for better flow. | 94 | The paragraph has been moved as suggested by you. |
|  | **Pre-planning** |  |  |
| 20 | Be mindful of emotive emphasis. A less loaded questions would be: Is it true that care of older dependent adults create/intensify financial burdens for their families? | 104 | We agree that it should be modified, many thanks for your advice. The question has been reformulated, following what you suggested. |
| 21 | What does this refer to? | 107 | This sentence has been changed to underline the contribution of the ScR to the mapping of concepts and detectable omissions. |
| 22 | Term used unclear | 110 | Changed. |
| 23 | Unclear – consider rephrasing | 114 | The sentence has been rephrased. |
|  | **Brainstorming** |  |  |
| 24 | impacted(?) | 125 | Changed as suggested by you. |
| 25 | Older adults or their caregivers/family? | 126 | The question has been rewritten to be more clearly addressed to older care recipients and their families. |
|  | **Definitive aims** |  |  |
| 26 | It is not clear to me what you mean here | 131 | The sentence has been changed to better underline the connection with the concepts used in literature and included in the conceptual framework. |
|  | **Initial keywords** |  |  |
| 27 | Here you describe what you have done in the past tense | 139 | The sentence has been changed. |
|  | **Protocol** |  |  |
| 28 | Here you describe what you have done in the present tense |  | The paragraph has been checked and changed using the present tense. |
| 29 | Do you want to refer to the final protocol by providing a link? Perhaps state it like this: “The final protocol of our study was submitted to…. and can be accessed via [provide link]”. | 151 | The paragraph has been changed according to your suggestion. |
|  | **Selection process and eligibility criteria** |  |  |
| 30 | This creates an interesting conundrum as research from the low- and middle-income countries may not be translated into English. With the funding available for this project, it may be good to reconsider this aspect to ensure a much more inclusive stance for relevant research? | 213 | The authors thank you for this advice and agree with you on the relevance of the local literature to the topic. The authors also agree that this problem represents one of the key issues for conducting a literature review. Unfortunately, within this project we are unable to conduct a broader search with a different language, because local and mother-tongue teams must be involved to carry it out, and the available budget and timetables do not allow it. For these reasons, the authors decided to adopt the most common strategy in literature review studies, and to focus on English literature only, as this is the most widely used scientific language. Considering also the relevance and the scarce diffusion of similar studies on these themes, the authors hope that this study could stimulate future studies including literature in other languages. These relevant suggestions have been used to integrate the text into the main file, aiming to explain the reasons of this limitative choice. |
|  | **Scoping review and evidence source details** |  |  |
| 31 | With a focus on quantitative studies, it is unclear why a systematic rather than scoping review was conducted | 226 | The choice relating to the kind of review to be carried out has been debated in the preliminary phases of the study design, as detailed in the paragraphs “Pre-planning” and “Brainstorming”. According to what is proposed by the guidelines adopted (Lockwood et al. 2019; Munn et al. 2018), independently of using quantitative or qualitative data - if the research questions are open and not too specific - it is recommended that researchers carry out a ScR, rather than a systematic review (see the seven aims listed in Fig. 2). The need to better understand the concepts involved in the conceptual framework and to understand how literature uses them, led us to start our reflection with a ScR. However, conducting a scoping review does not exclude that a systematic review can be carried out on the same issue in the future. |
| 32 | Why? Please explain as there could be mixed methods to illuminate quantitative findings. | 229 | Many thanks for this advice. In this section we explain the different data sources. The useful contribution to a better understanding of phenomena coming from the integrated use of both quantitative and qualitative studies has been underlined by an additional sentence. |
|  | **Conducting and reporting** |  |  |
| 33 | Examined? Screened? | 271 | The word has been changed into “screened” as suggested by you. |
|  | **Initial selection outputs** |  |  |
| 34 | Be mindful of switching between past and present tense. | 275 | We checked the text again. |
|  | **Quality assessment: Data extraction** |  |  |
| 35 | Please consider the focus of scoping reviews and that this usually do not comment on the quality of the research as it aims to provide an overview (the whole scope) of research related to a specific topic area | 301 | Many thanks for your advice. What we assessed here was not the quality of research but methods reporting, i.e. how the methods chosen have been described. The sentence in the text has been changed to better clarify this aim. |
| 36 | Contradicts to what was stated above? | 319 | The data analysis section has been revised and integrated to underline the mixed method analysis to be used in this study: descriptive quantitative analysis (based on frequency analysis) and contents qualitative analysis (to explore contents of results). |
